# Performance Modeling of Spherical Capsules during Mixing of Self-Consolidating Concrete

**DOI:** 10.3390/ma16062379

**Published:** 2023-03-16

**Authors:** Samir E. Chidiac, Mouna A. Reda

**Affiliations:** Department of Civil Engineering, McMaster University, 1280 Main Street West, Hamilton, ON L8S 4L7, Canada

**Keywords:** self-healing, capsule survival rate, FE model, probability, rheological properties, concrete, concrete pan mixer

## Abstract

Autonomous healing is a very promising technique in self-healing concrete systems. For capsules to achieve their anticipated performance, they should be able to survive the harsh mixing conditions of concrete, yet rupture upon concrete cracking. At present, there are no standard test methods, either experimental or analytical, for determining the capsule survival rate during concrete mixing. This study investigates the correlation between the capsules’ shell properties, concrete rheological properties, the capsules’ external forces, and capsule survival rate during concrete mixing. Finite element and statistical modeling techniques were employed to evaluate the capsule performance and predict the survival rate of capsules during concrete mixing, with 68% confidence. The results revealed that the capsules’ survivability during concrete mixing is highly influenced by the capsule’s radius-to-thickness ratio, the rheological properties of the fresh concrete, the average-paste-thickness (APT) of the concrete mix, the aggregate content and angularity, and the speed of the mixer. In brief, capsules with a radius-to-thickness ratio between 30 and 45 are likely to survive concrete mixing and yet still rupture upon concrete cracking.

## 1. Introduction

Encapsulation, which is employed to protect the healing agent during the mixing and casting of fresh concrete and to release the agent upon the cracking of hardened concrete [1,2], is a promising technique for the creation of autonomous self-healing concrete systems [3,4,5,6,7,8,9,10,11,12,13,14,15,16,17,18,19,20,21,22]. For the capsules to achieve their objective, the shell’s mechanical and geometrical properties need to be compatible with those of the concrete matrix. Ideally, the shell needs to be ductile to endure the harsh concrete mixing conditions and brittle to rupture upon concrete cracking. Different shell materials, including glass [5,21,22,23,24,25,26,27,28,29], ceramics [21,29], and polymers [2,3,4,9,12,14,15,16,18,19,30,31,32,33,34,35,36,37,38,39,40,41], have been investigated and tested in the literature. Test results revealed that glass and ceramics have low survivability during mixing [42,43], whereas polymers have “switchable” mechanical properties, with a higher survival ratio [13,44,45,46,47]. The robustness of capsules to survive mixing conditions requires not only their resistance to the shear forces applied by the concrete mixer, but also to the punching stress exerted by the aggregates [47,48,49]. The review of the literature revealed that there are no standard test methods for measuring the performance of capsules in terms of survivability during concrete mixing and placing. The studies documented in the literature, which are presented here, show inconsistencies in the results, as different test methods and measuring techniques are used to assess performance. Moreover, the survival rate of the capsules during mixing is found to be highly influenced by the geometrical and mechanical properties of the shell, the concrete rheological properties, and the speed and type of the concrete mixer. As such, there is a need to develop standard testing protocol to evaluate the performance of capsules in self-healing cementitious materials during the mixing and placing of concrete, as their performance impacts the efficiency and efficacy of self-healing concrete systems, as well as the subsequent hardened properties of concrete thereafter. This study aims to address this need by analytically investigating the relationship between the shell’s geometrical and mechanical properties, concrete rheological properties, and the survival rate of the self-healing capsules. Numerical and statistical models are employed to predict the probability of capsule survival during mixing, with 68% confidence. This paper includes five parts: the introduction; a brief literature review of the test methods used to evaluate the survivability of capsules during concrete mixing; the methodology proposed to determine the survivability of capsules in concrete during mixing, which includes material properties, an idealized model of capsules during mixing, and the design of the experiment; the model results, along with an analysis and discussion of these results; and the conclusions.

## 2. Literature Review—A Brief

The survivability of capsules during concrete mixing is pivotal to the effectiveness of self-healing concrete systems. As the vessel for the healing agents, if the capsules were ruptured during mixing, then their presence in the self-healing cementitious matrix becomes detrimental to the mechanical properties of the concrete matrix. Among several encapsulation materials investigated in the literature for their suitability in self-healing concrete applications, glass and ceramics have shown limited ability to survive mixing conditions, both requiring protective techniques to enhance their ductility [25,29,47,48,50,51], while polymeric materials have shown promising results in surviving concrete mixing [13,44,45,46,47].

The review of the literature revealed two testing methodologies that have been adopted to investigate the survivability of self-healing capsules: (1) chemical stability [3,13,38,45,46,49,52,53,54], and (2) mechanical stability [22,44,55,56]. The survivability of the capsules is determined by either manually counting the number of intact capsules and/or by visually inspecting the capsules’ morphology using an optical microscope and scanning electron microscopy (SEM) [16,31,57,58,59].

The chemical stability test, which consists of immersing the capsules in a high pH solution, mimicking concrete pore solution, followed by a mechanical test, aims to ensure that the capsules’ mechanical properties are not altered by the concrete pore solution. The tensile strength of PLA, PS, and P(MMA-n-BMA) tubes was tested before and after immersing them in cement slurry with a pH of 12.5 to 13 for 7 to 14 days. The results revealed that only the P(MMA/n-BMA) tubes’ strength was lower [3,53]. Other researchers investigated the chemical resistance of soda glass capsules by filling the capsules with a traceable fluorescent dye and immersing them in a solution with a pH of 12. They reported no leakage or observed change in the physical properties [52]. Lv et al. [38] and Giannaros et al. [49] immersed phenol-formaldehyde (PF) dicyclopentadiene (DCPD)-filled microcapsules and sodium silicate-filled poly-urea and gelatin-gum arabic capsules, respectively, in saturated calcium hydroxide solution for 48 h, and confirmed through SEM images that the capsules maintained their morphology. Kanellopoulos et al. [13] and Al-Tabbaa et al. [46] tested the chemical stability of dry and hydrated gelatin-acacia gum microcapsules by immersing them for 2 months in sodium hydroxide solution with a pH of 11.5, 12.5, and 13.5. Using SEM and optical microscope images, the capsules were found to remain spherical. Moreover, Mao et al. [54] confirmed the survivability of the sodium silicate microcapsules with a polyurea shell for oil well cement application by immersing the capsules in a saturated calcium hydroxide solution with a pH of 13 at 80 °C for 14 days, and other capsules in a cement slurry. SEM images of the capsules in the saturated solution or the thin cement paste layer extracted from the cement slurry showed that the capsules survived the mixing process.

Testing the mechanical stability during mixing was reported for large-size capsules, with a visual count employed to determine the number of intact capsules. The developed tests consist of mixing the fresh concrete, then sieving it under running water to capture the surviving capsules. Hu et al. [22] mixed 10 glass cylindrical capsules with an 8 mm inner diameter, 1 mm thickness, and 30 mm length, and filled with polyurethane healing agent, with cement mortar in a mixing pan at a speed of 65 rpm for 3 min and reported a survival rate of 90 to 100%. Gruyaert et al. [55] mixed 10 ethyl cellulose (EC) cylindrical capsules with a 3 mm inner diameter, 1 mm thickness, and 50 mm length, and 10 glass EC-coated capsules with a 1.7 mm inner diameter, 0.3 mm thickness, and 25 to 50 mm length, using 3 different concrete batches in a concrete mixer for 2 min at 140 rpm, followed by 1 min at 285 rpm. The results revealed a 100% capsule survival when 25% plasticizer was used in the mix, a 40 to 90% survival with 10% plasticizer for EC capsules, and an 80-90% survival for glass-EC coated capsules. Sinha et al. [56] mixed 20 polylactic acid (PLA)-based biomass elongated elliptical capsules with 12 concrete batches in a revolving drum tilting mixer. To account for the effect of the capsules’ geometry, a range of sizes between 5 to 19.05 mm and thicknesses of 0.4 to 2 mm was used, with aspect ratio varying between 1:1:1, 1.5:1:1, and 2:1:1, to mimic gravel and sand sizes used in the concrete mix. The standard concrete mixing protocol was first used, then the capsules were added to the mix and mixed for an additional 5 min. Capsules of a smaller size and a higher aspect ratio were found to perform better; specifically, the survival ratio of the 9.5 mm capsules with an aspect ratio of 2:1:1 and a 0.4 mm thickness was 95 to 100%. Araújo et al. [44] mixed the dry components of concrete for 1 min, added the water, and mixed for another 1 min, then added the poly (methyl methacrylate) capsules and mixed the mixture for 2 additional min. Three sets of cylindrical capsule sizes and thicknesses were used: 6.5 ± 0.3 mm, 0.7 ± 0.1 mm, 5.9 ± 0.6 mm, 0.4 ± 0.1 mm, and 5.8 ± 0.3 mm, 0.2 ± 0.1 mm, with a 50 mm length. They concluded that the capsules with thicker walls have a higher survival rate.

From the previous studies, the following observations can be made:

- Standardized tests to evaluate the survivability of capsules during concrete mixing are needed.
- Test results show that capsules have the required chemical resistance to survive in a high alkaline concrete pore solution.
- Capsule survival rate, according to chemical and/or mechanical tests, is deduced from visual inspection of a few capsules and assuming that the sample is representative of the whole sample. The statistical properties of the sample must be established before accepting such an approach.
- The type of mixer, speed of mixing, mixing time, and mixing technique varied between different studies, which makes it difficult to compare the results, even for the capsules made with the same materials.
- Concrete rheological properties affect the capsule survivability rate.
- Capsules in mortar have a higher survivability than those added to concrete.
- Capsules with a smaller diameter and thicker walls have a higher survival rate.

## 3. Methodology

Finite element and statistical modeling techniques were employed for determining the survivability of polymeric capsules in a concrete mixer. Experiments carried out by the authors and data reported in the literature were used to quantify the rheological properties of fresh concrete and the mechanical and geometrical properties of polymeric capsules, respectively. It was postulated that the rheological properties of concrete and the rotational speed of the mixer affect the shearing stress and normal stress exerted on the capsules during mixing, respectively. Standard deviation was the measurement used to account for the variability in the capsules’ geometrical and mechanical properties. The interactions between the input variables and the capsule survival rate were predicted with 68% confidence. Details of the material properties, model development, and design of the experiments are provided next.

### 3.1. Material and Geometrical Properties

Two self-consolidating concrete (SCC) mixes, designed to have a minimum slump flow of 600 mm, were used in this study. A ring-pan mixer [60] was used for mixing the SCC mixes. The composition and measured properties of the two mixes are given in Table 1. Portland-limestone cement (CSA type GUL) and ground granulated blast furnace slag (GGBFS) were provided by Lafarge Holcim, Canada. The corresponding physical and chemical properties are presented in Table 2. High-range water-reducing admixture (HRWRA), Glenium© 7700 [61], and viscosity modifying admixture (VMA), MasterMatrix© VMA 362 [62], were added to the mixes to achieve the design slump flow. Regarding the coarse aggregates, the nominal maximum aggregate size, specific gravity, bulk density, and absorption are 14 mm, 2.74, 1544 kg/m^3^, and 1.58%, respectively. The fineness modulus, specific gravity, bulk density, and absorption of fine aggregate are 2.88, 2.71, 1746 kg/m^3^, and 1.28%, respectively. The bulk density, specific gravity, and absorption of coarse and fine aggregates were determined in accordance with ASTM C127-15 [63] and ASTM C128-15 [64], respectively. The slump flow and density were measured in accordance with ASTM C1611-18 [65] and CSA A23.2-4C:14 [66], respectively. RheoCAD 500, a concrete rheometer developed by CAD Instruments [67], along with the Bingham material model [68,69,70], were used to estimate the rheological properties of the SCC mixes.

For the capsule, two values for the diameter and thickness were considered, along with a single value for the rupture strength and fracture energy, as given in Table 3. Standard deviation, which was estimated from the work of Wang et al. [9], was included to account for the variability in the capsules’ geometry and rupture strength. The capsules’ rupture strength and fracture energy were deduced from data reported in the literature for the polymeric shell material [72].

### 3.2. Finite Element Model

**Figure 1:**
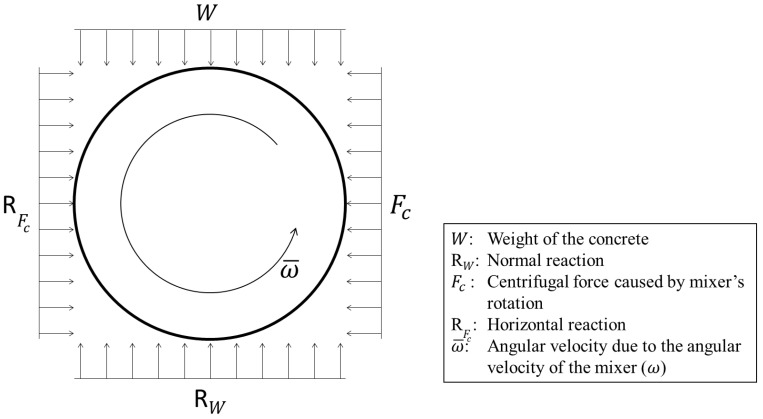
Free-body diagram of a capsule in a rotating concrete mixer.

The model consists of a single capsule located at the bottom and outer edge of the concrete mixer. The free-body diagram of the capsule in a rotating concrete mixer is shown in Figure 1. This scenario afforded the inclusion of all the external forces exerted on the capsule, namely the weight of the concrete (W), the centrifugal force ($F_{c}$) caused by the rotation of the mixer, and the shear force (τ) due to mixing and flow of fresh concrete.

The weight (W) corresponding to the concrete layer atop the capsule can be estimated by

(1) $$W=\rho \frac{\pi }{4}D_{s}^{2}t_{c}g$$

in which *ρ* is the density of the fresh concrete (kg/m^3^), $D_{s}$ the diameter of the capsule (m), $t_{c}$ the thickness of the concrete layer atop the capsule (m), and g the gravitational acceleration (m/s^2^). The thickness of the concrete layer is deduced from the size of the mixer and its maximum concrete yield.

The centrifugal force ($F_{c}$) caused by the rotation of the mixer is estimated by

(2) $$F_{c}=\rho \frac{\pi }{4}D_{s}^{2}{\left(\frac{d_{m}}{2}\right)}^{2}\omega^{2}$$

in which *d_m_* is the diameter of the mixer (m), and ω the angular velocity of the mixer (rad/s). For the LIEBHERR ring-pan mixer Type R [60] used in this study, the nominal capacity, outer diameter, and rotational velocity of the mixer are 1 m^3^ of concrete, 2.425 m, and 26 rpm, respectively.

The forces, W and $F_{c}$, can be applied over the surface area of the capsule or as a point force. The former represents the contact pressure between the concrete paste and the capsule, and the latter represents the punching of the capsule by an aggregate. The contact surface is modeled in accordance with Hertzian contact pressure, where

(3) $$P\left(r\right)=P_{o}{\left(1-\frac{r^{2}}{D_{s}^{2}}\right)}^{\frac{1}{2}}$$

in which $P_{o}$ is the maximum contact pressure corresponding to W and $F_{c}$ over the surface area of the capsule (MPa), r the radial distance (m), and $D_{s}$ the diameter of the capsule. The Hertzian contact model has been used to characterize the response of microcapsules tested under compression in several self-healing studies [73,74,75].

The likelihood of an aggregate punching a capsule, increases with the decrease in concrete paste thickness. As such, a model developed by Chidiac et al. [76] is employed to determine the average cement paste thickness (APT) of the 2 SCC mixes. The APT model is reproduced below, where

(4) $$APT=-\frac{1}{2}\left(D_{fa}+\frac{\phi_{ca}D_{fa}^{2}}{\phi_{fa}D_{ca}}+\frac{\phi D_{fa}^{2}\left(1-\phi_{max}\right)}{\phi_{fa}\phi_{max}D}\right)+\frac{1}{2}\sqrt{{\left(D_{fa}+\frac{\phi_{ca}D_{fa}^{2}}{\phi_{fa}D_{ca}}+\frac{\phi D_{fa}^{2}\left(1-\phi_{max}\right)}{\phi_{fa}\phi_{max}D}\right)}^{2}+\frac{4}{3}\frac{\left(\phi_{max}-\phi \right)}{\phi_{max}}\frac{D_{fa}^{2}}{\phi_{fa}}}$$

(5) $$\mathrm{and}\;D={\left(\frac{D_{ca}{}^{3}\phi_{ca}+D_{fa}{}^{3}\phi_{fa}}{\phi_{ca}+\phi_{fa}}\right)}^{1/3}$$

in which D, $D_{fa}$, and $D_{ca}$ are the mean diameters of the total aggregate gradation, fine aggregate gradation, and coarse aggregate gradation corresponding to 50% passing, respectively. ϕ, $\phi_{fa}$, $\phi_{ca}$, and $\phi_{max}$ are the volume fraction of the aggregates, fine aggregates, coarse aggregates, and maximum packing density of aggregates, respectively. The volume fraction of the fine and coarse aggregates, along with $\phi_{max}$, $\phi /\phi_{max}$, D, and APT for the 2 mixes are reported in Table 4. The results, which show that the capsule diameter is significantly larger than the APT, indicate that the likelihood of the capsules being punched by the aggregates is high.

The mixing of fresh concrete begins when the shear stress (τ) exceeds the concrete yield stress ($\tau_{0}$) and increases linearly with the concrete plastic viscosity (μ) and shear strain rate ($\dot{\gamma }$), according to the Bingham model [68,69,70], where

(6) $$\tau =\tau_{0}+\mu \dot{\gamma }$$

The concrete Bingham properties represented by the yield stress and plastic viscosity were measured experimentally and are reported in Table 1. The shear strain rate ($\dot{\gamma }$) is assumed to be uniform and is calculated using the following relationship:

(7) $$\dot{\gamma }=\frac{\omega \left(\frac{d_{m}}{2}\right)}{t_{c}}$$

**Figure 2:**
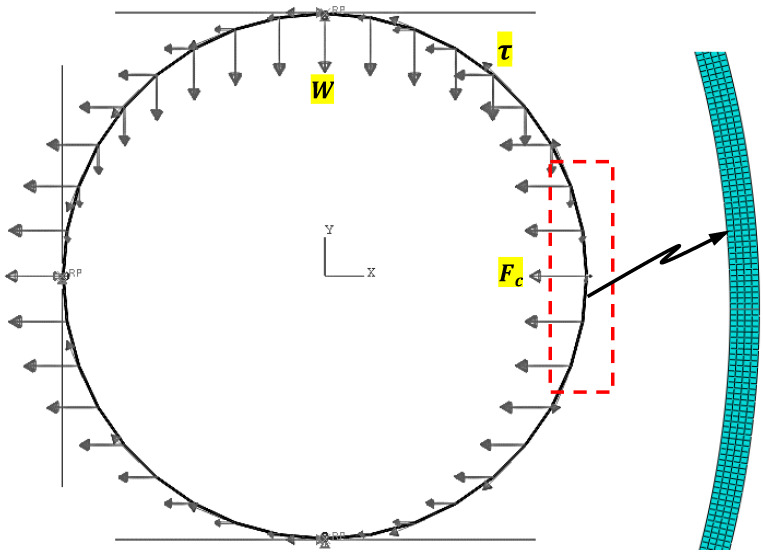
FE model includes geometry, boundary and loading conditions, and mesh.

An idealized 2-D plane strain finite element (FE) model was developed for this analysis using the commercial finite element program ABAQUS [77]. The FE model, which is shown in Figure 2, consists of a single spherical capsule surrounded by three contact surfaces representing the bottom and side edge of the mixer and the concrete atop the capsule. The contact interface between the mixer wall and the capsule outer layer is modeled as surface friction, with a static friction coefficient of 0.34 [78]. The top contact interface represents the cohesive structure of the fresh concrete atop the capsule. The three loads shown, i.e., vertical, horizontal, and circumferential, represent the weight of the concrete atop the capsule, the centrifugal force, and the shear force, respectively. The FE mesh consists of 8-node biquadratic plane strain quadrilateral elements (CPE8), with 4 elements along the thickness of the capsules, with a 1:1 aspect ratio.

The cohesive interface surface model, developed by ABAQUS [77], was employed to capture the fracture of the capsule surface. The interface was placed along the horizontal direction passing through the diameter of the shell. Damage initiates when the nominal stress reaches the rupture strength of the capsule and propagates based on the energy dissipation governed by the fracture toughness (G) [72].

### 3.3. Design of Experiments

The numerical experiments were designed to determine the interactions between the input parameters and the output, specifically, the survival of the capsules. Two diameters and two thicknesses of the capsule were included. The mean plus and minus one standard deviation were analyzed for the capsule geometry and the capsule rupture strength. Moreover, two loadings were considered to account for the interactions between the capsules and the concrete paste, and the capsules and the aggregates. The analyses executed are summarized in Table 5.

## 4. Results, Analysis, and Discussion

**Figure 3:**
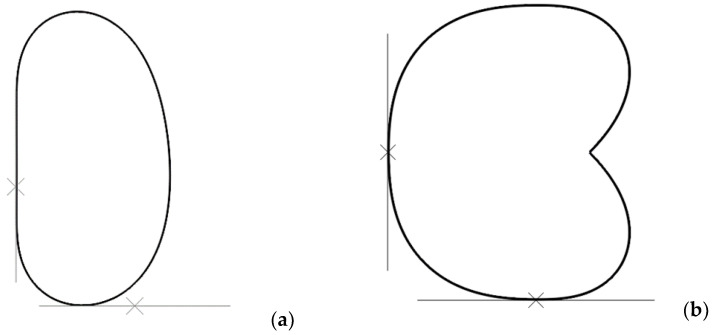
Capsule failure modes deduced from the FE analysis: (**a**) stretching and/or rupturing; (**b**) punching.

The results revealed two potential failure modes for the capsule, as shown in Figure 3. The first mode is due to the stretching and/or rupturing of the capsule shell caused by the pressure from the surrounding concrete paste, and the second mode is due to the punching of the capsule shell caused by the aggregates. The criteria for the first failure mode are the elongation of the shell and the rupture strength of the material. The criterion for the second failure mode is the rupture strength of the material. For reference, the rupture strength of the capsule is f_rs_ = 30.0 ± 2.5 MPa, and the elongation limit of the urea-formaldehyde shell is 0.75 ± 0.08% [79].

The FE analyses, which yielded the shell state of the stress and strain, as well the survivability of the capsule, when subjected to squeezing and stretching by concrete paste and pinching by aggregates, are summarized in Table 6. CSMAXSCRT is a variable in ABAQUS that indicates whether the contact stress damage initiation criterion has been satisfied at the contact point, with a value of 0 for an undamaged surface to 1 for the initiation of damage [77]. The results also include the maximum contact pressure, the maximum true logarithmic strain, and the observed failure mode.

Assuming the results follow a normal distribution, the probability of capsule failure based on a 68% confidence level was analyzed using the finite element results and the corresponding material properties. The three failure modes were analyzed separately, and the results, in the form of probability of failure (P_f_), are presented in Table 7. The overall P_f_ was determined on the assumption that 90% of the capsules are surrounded by concrete paste and 10% by aggregates. From the results of Table 7, the probability of failure of the two capsule sizes, specifically 0.2 mm and 2.0 μm, and 0.5 mm and 3.0 μm, for mixes 1 and 2, are 6.6%, 11.2%, 8.7%, and 11.3%, respectively. Recalculating the overall P_f_, with the assumption that 100% of the capsules are surrounded by concrete paste and 0% by aggregates, the results become 0.0%, 1.3%, 2.6%, and 1.9%, respectively. These results reveal that (1) the rheological properties of the concrete mix affect the survivability of small size capsules and that the effect decreases with an increase in capsule diameter; (2) the survivability of the capsule decreases as the capsule size increases, regardless of the rheological properties of the concrete; (3) the interaction of aggregates with the capsules significantly affects the survivability of the capsules; and (4) on average, 90% of the capsules are expected to survive the mixing of concrete, given a material rupture strength of 30 ± 2.5 MPa and elongation limit of 0.75 ± 0.08%.

**Figure 4:**
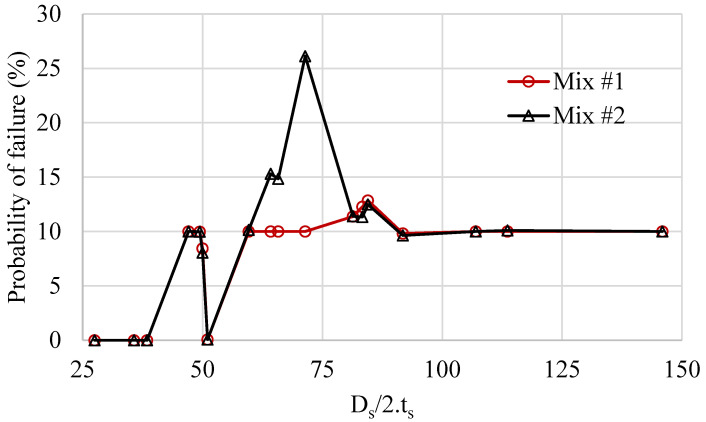
Capsule radius-to-thickness probability of failure.

Figure 4 displays the probability of failure calculated for the capsules’ radius to thickness for both concrete mixes. The effects of the capsules’ geometry and rheological properties of the concrete mix are evident. The results show that for capsules whose radius-to-thickness ratio is greater than 45, their probability of failure increases from 0 to 10%. Moreover, the combined effect of the concrete rheological properties and the capsules’ geometry on the capsules’ survivability is complex. For mix #2, an increase from 10% to 26% is observed for the capsules whose radius-to-thickness ratio range between 60 and 80, and an increase from 10% to 13% is observed for both mixes for the capsules whose radius-to-thickness ratio range between 80 and 90. In general, the capsules’ survivability decreases to 90% once the capsule radius-to-thickness ratio is greater than 45. This finding agrees well with a previous recommendation for capsule radius-to-thickness ratio ranging between 30 and 100 for rupturing instead of debonding upon the cracking of hardened concrete [72]. Based on these results, the recommendations are amended to a capsule radius-to-thickness ratio of 30 to 45 for surviving concrete mixing, yet still rupturing upon the cracking of hardened concrete.

The FE results are found to agree with Souza and Al-Tabbaa experimental data [59]. Their SEM images revealed that the acrylate shell of the capsules’ radius-to-thickness ratio of 6.3 and 27.5 survived concrete mixing. Kanellopoulos et al. [13] used Gelatin-acacia gum capsules and reported that capsules whose radius-to-thickness of about 50 debonded from the cement paste prisms after testing under a three-point-bending test, while the smaller capsules whose radius-to-thickness less than 30 showed better bonding with the surrounding matrix [13]. These experimental findings further support our amended recommendation of using capsules whose radius-to-thickness ratio is between 30 to 45 to ensure their survivability during concrete mixing and yet still rupture upon cracking of hardened concrete.

## 5. Conclusions

This paper presents a finite element model for studying the survivability of capsules during concrete mixing. The model considers all possible interactions between the capsules and the surroundings, as well as accounts for the geometry of the capsules and the rheological properties of the concrete. The probabilities of capsule failure were determined using statistical models and assuming a normal distribution. Accordingly, the following conclusions are drawn:

1. Research studies on the survivability of self-healing capsules during concrete mixing are found to be lacking despite its significance to the performance of self-healing concrete system.
2. The capsules’ radius-to-thickness ratio highly influences the survivability of the capsules during concrete mixing.
3. The rheological properties of fresh concrete affect the survivability of small-sized capsules, and that effect decreases with an increase in capsule diameter.
4. The interaction between the aggregates and the capsules adversely affects the survivability of the capsules.
5. In capsules whose radius-to-thickness ratio is greater than 45, the probability of failure increases from 0 to 10%.
6. The combined effect of the concrete’s rheological properties and capsules’ geometry on the capsules’ probability of failure increased from 10% to 26% for mix #2 when the capsules’ radius-to-thickness ratio was between 60 and 80, and from 10% to 13% for both mixes when the capsules’ radius-to-thickness ratio was between 80 and 90.
7. The survivability of the capsule decreases to 90% when the capsules’ radius-to-thickness ratio is greater than 90, regardless of the concrete rheological properties.

The recommendations are to design self-healing concrete system with a capsule radius-to-thickness ratio between 30 to 45 to survive concrete mixing and yet still rupture upon the cracking of hardened concrete, and that a standard test should be developed to account for the capsule geometry and material properties; the concrete rheological properties; the concrete mix design, specifically the APT, the aggregate content, and the angularity; and the speed of the mixer. Lastly, the findings, conclusions, and recommendations are specific to the variables investigated in this study.

## Figures and Tables

**Table 1:** Self-consolidating concrete mix design used in the FE model.

Mixture Proportion	SCC Mix #1	SCC Mix #2
Water-to-Cementing Materials Ratio, w/cm	0.32	0.32
GUL (% mass of cm)	100	70
GGBFS (% mass of cm)	0	30
Cementing Content (kg/m^3^)	450	450
Volume Fraction of Coarse Aggregate, V_CA_ (m^3^/m^3^)	0.30	0.25
Volume Fraction of Fine Aggregate, V_FA_ (m^3^/m^3^)	0.35	0.40
HRWRA, (% mass of cm)	0.84	0.69
**Fresh Properties**		
Density, ρ (kg/m^3^)	2451	2416
Slump Flow, S_f_ (mm)	638	680
Viscosity, μ (Pa.s)	49	78
Yield Stress, τ_0_ (Pa)	40	16

**Table 2:** Physical and chemical properties of cement used in the study [71].

Oxides, Compounds	Composition (% Mass)	
	GUL	GGBFS
CaO	61.3	36.9
SiO_2_	18.0	36.2
Al_2_O_3_	4.4	10.4
Fe_2_O_3_	2.8	0.6
MgO	2.9	11.9
K_2_O	0.5	0.5
Na_2_O	0.2	0.4
Na_2_O_eq_	0.6	0.8
SO_3_	3.6	2.7
TiO_2_	0.3	1.1
MnO	2.9	0.5
Free CaO	1.1	
Limestone	11.5	
Loss on Ignition	5.5	0.8
Total	96.8	101.2
C_3_S	47	
C_2_S	16	
C_3_A	7	
C_4_AF	8	
Specific Surface Area, Blaine (m^2^/kg)	468	475
Specific Gravity	3.15	2.92
Compressive Strength, 28d (MPa)	41.7	

**Table 3:** Capsule properties used in the FE model.

Variables	Mean ± Standard Deviation	
Diameter of Capsule, D_s_ (mm)	0.2 ± 0.057	0.5 ± 0.142
Shell Thickness, t_s_ (mm)	0.002 ± 0.0006	0.003 ± 0.0008
Rupture Strength of Capsule, f_rs_ (MPa)	30.0 ± 2.5	
Fracture Energy, G_s_ (J/m^2^)	100	

**Table 4:** Aggregate volume fractions and APT measurements.

Mix #	*ϕ_fa_*	*ϕ_ca_*	*ϕ_max_*	*ϕ*/*ϕ_max_*	D (mm)	APT (mm)
1	0.35	0.30	0.78	0.83	6.56	0.11
2	0.40	0.25	0.77	0.84	6.18	0.10

**Table 5:** Design of experiment developed for this study.

Loading Scenario #1: Hertz Contact Pressure							
*D_s_* (mm)	*t_s_* (mm)	Mix #1			Mix #2		
		Centrifugal Stress (kPa)	Weight (kPa)	Shear Stress (kPa)	Centrifugal Stress (kPa)	Weight (kPa)	Shear Stress (kPa)
0.200 ± 0.057	0.002 ± 0.0006	8.68	1.69	1.02	8.56	1.67	1.57
0.500 ± 0.142	0.003 ± 0.0008						
**Loading Scenario #2: Aggregate Punching**							
0.200 + 0.057	0.002 ± 0.0006	1.796	1.69	1.02	1.771	1.67	1.57
0.200	0.002 ± 0.0006	1.091			1.075		
0.200 − 0.057	0.002 ± 0.0006	0.561			0.553		
0.500 + 0.142	0.003 ± 0.0008	11.226			11.066		
0.500	0.003 ± 0.0008	6.818			6.721		
0.500 − 0.142	0.003 ± 0.0008	3.504			3.454		

**Table 6:** FE results including observed capsule performance.

Mix #1		Loading Scenarios							
*D_s_* (mm)	*t_s_* (μm)	Contact Pressure					Point Load		
		CSMAXSCRT	Pressure (MPa)	Performance	Log Strain (%)	Performance	CSMAXSCRT	Pressure (MPa)	Performance
0.257	1.4	0.8	24.27	Survived	0.55	Survived	1.0	34.11	Ruptured
0.257	2.0	0.6	17.83	Survived	0.50	Survived	1.0	42.11	Ruptured
0.257	2.6	0.4	12.60	Survived	0.31	Survived	1.0	37.75	Ruptured
0.200	1.4	0.7	20.21	Survived	0.59	Survived	1.0	41.68	Ruptured
0.200	2.0	0.4	12.81	Survived	0.32	Survived	0.9	32.53	Survived
0.200	2.6	0.3	8.07	Survived	0.19	Survived	0.5	18.64	Survived
0.143	1.4	0.4	13.27	Survived	0.33	Survived	0.7	23.87	Survived
0.143	2.0	0.2	7.02	Survived	0.17	Survived	0.3	11.58	Survived
0.143	2.6	0.1	4.19	Survived	0.10	Survived	0.2	7.026	Survived
0.642	2.2	0.5	16.22	Survived	0.54	Survived	1.0		Ruptured
0.642	3.0	0.8	23.13	Survived	0.48	Survived	1.0		Ruptured
0.642	3.8	0.8	25.36	Survived	0.61	Survived	1.0	41.19	Ruptured
0.500	2.2	0.7	19.52	Survived	0.44	Survived	1.0		Ruptured
0.500	3.0	0.9	26.49	Survived	0.60	Survived	1.0	37.22	Ruptured
0.500	3.8	0.6	19.20	Survived	0.53	Survived	1.0	44.62	Ruptured
0.358	2.2	0.9	26.02	Survived	0.59	Survived	1.0	36.52	Ruptured
0.358	3.0	0.6	16.81	Survived	0.45	Survived	1.0	51.59	Ruptured
0.358	3.8	0.4	11.76	Survived	0.29	Survived	1.0	52.44	Ruptured
**Mix #2**		**Loading Scenarios**							
***D_s_*** **(mm)**	***t_s_*** **(μm)**	**Contact Pressure**					**Point Load**		
		**CSMAXSCRT**	**Pressure** **(MPa)**	**Performance**	**Log Strain (%)**	**Performance**	**CSMAXSCRT**	**Pressure** **(MPa)**	**Performance**
0.257	1.4	0.8	24.39	Survived	0.55	Survived	1.0	33.74	Ruptured
0.257	2.0	0.9	26.09	Survived	0.76	Ruptured	1.0	41.42	Ruptured
0.257	2.6	0.6	18.24	Survived	0.47	Survived	1.0	37.25	Ruptured
0.200	1.4	0.9	27.71	Survived	0.70	Ruptured	1.0	36.31	Ruptured
0.200	2.0	0.6	18.56	Survived	0.48	Survived	0.9	32.15	Survived
0.200	2.6	0.4	12.02	Survived	0.29	Survived	0.5	18.48	Survived
0.143	1.4	0.6	19.19	Survived	0.50	Survived	0.7	23.69	Survived
0.143	2.0	0.4	10.55	Survived	0.25	Survived	0.3	11.46	Survived
0.143	2.6	0.2	6.37	Survived	0.15	Survived	0.2	6.964	Survived
0.642	2.2			Survived		Survived	1.0		Ruptured
0.642	3.0			Survived		Survived	1.0		Ruptured
0.642	3.8	0.9	27.10	Survived	0.61	Survived	1.0	34.55	Ruptured
0.500	2.2	0.7	22.28	Survived	0.76	Ruptured	1.0	31.96	Ruptured
0.500	3.0	0.9	26.76	Survived	0.60	Survived	1.0	33.41	Ruptured
0.500	3.8	0.9	25.98	Survived	0.74	Ruptured	1.0	43.74	Ruptured
0.358	2.2	0.9	26.09	Survived	0.59	Survived	1.0	43.17	Ruptured
0.358	3.0	0.8	22.67	Survived	0.65	Survived	1.0	55.53	Ruptured
0.358	3.8	0.6	17.01	Survived	0.44	Survived	1.0	51.42	Ruptured

**Table 7:** Probabilities of capsule failure.

Mix #1			Probability of Failure (%)			
*D_s_* (mm)	*t_s_* (μm)	D_s_/2t_s_	Contact Pressure		Point Load	Total
			Rupturing	Stretching	Rupturing	
0.257	1.4	92	1.1	0.4	95.0	9.8
0.257	2.0	64	0.0	0.1	100.0	10.0
0.257	2.6	49	0.0	0.0	99.9	10.0
0.200	1.4	71	0.0	1.6	100.0	10.0
0.200	2.0	50	0.0	0.0	84.4	8.4
0.200	2.6	38	0.0	0.0	0.0	0.0
0.143	1.4	51	0.0	0.0	0.7	0.1
0.143	2.0	36	0.0	0.0	0.0	0.0
0.143	2.6	28	0.0	0.0	0.0	0.0
0.642	2.2	146	0.0	0.3	100.0	10.0
0.642	3.0	107	0.3	0.0	100.0	10.0
0.642	3.8	84	3.2	3.2	100.0	12.9
0.500	2.2	114	0.0	0.0	100.0	10.0
0.500	3.0	83	8.0	2.5	99.8	12.3
0.500	3.8	66	0.0	0.2	100.0	10.0
0.358	2.2	81	5.6	1.6	99.5	11.4
0.358	3.0	60	0.0	0.0	100.0	10.0
0.358	3.8	47	0.0	0.0	100.0	10.0
**Mix #2**			**Probability of Failure (%)**			
***D_s_*** **(mm)**	***t_s_*** **(μm)**	**D_s_/2t_s_**	**Contact Pressure**		**Point Load**	**Total**
			**Rupturing**	**Stretching**	**Rupturing**	
0.257	1.4	92	1.2	0.3	93.3	9.6
0.257	2.0	64	5.9	57.6	100.0	15.3
0.257	2.6	49	0.0	0.0	99.8	10.0
0.200	1.4	71	18.0	27.4	99.4	26.1
0.200	2.0	50	0.0	0.0	80.5	8.1
0.200	2.6	38	0.0	0.0	0.0	0.0
0.143	1.4	51	0.0	0.0	0.6	0.1
0.143	2.0	36	0.0	0.0	0.0	0.0
0.143	2.6	28	0.0	0.0	0.0	0.0
0.642	2.2	146	0.0	0.0	100.0	10.0
0.642	3.0	107	0.0	0.0	100.0	10.0
0.642	3.8	84	12.3	3.1	96.6	12.5
0.500	2.2	114	0.1	55.5	100.0	10.1
0.500	3.0	83	9.7	2.4	91.4	11.3
0.500	3.8	66	5.4	45.8	100.0	14.9
0.358	2.2	81	5.9	1.6	100.0	11.4
0.358	3.0	60	0.2	8.5	100.0	10.2
0.358	3.8	47	0.0	0.0	100.0	10.0
